# Effect of age on heart rate, blood lactate concentration, packed cell volume and hemoglobin to exercise in Jeju crossbreed horses

**DOI:** 10.1186/s40781-017-0126-8

**Published:** 2017-01-25

**Authors:** Ok-Deuk Kang, Yong-Soo Park

**Affiliations:** 1 Department of Hippotherapy Welfare, SungDuk C. University, Yeongcheon-si, 38801 Gyeongsangbuk-do Korea; 2 Department of Horse Industry, Korea National College of Agriculture and Fisheries, Jeonju-si, Jeollabuk-do 54874 Republic of Korea; 3 105, Daehak-gil, Sinnyeong-Myeon, Yeongcheon-si, Gyeongsngbuk-do 38801 Korea

**Keywords:** Age of horses, Animal welfare, Exercise test, Endurance, Physical response

## Abstract

**Background:**

This study aimed to analyze the on heart rate, blood lactate concentration, packed cell volume (PCV) and hemoglobin (Hb) response after conducting exercise in endurance horses.

**Methods:**

A total of 20 healthy 3–9-years-old Jeju crossbreed mares (5.95 ± 2.24 year) of age and 312.65 ± 13.59 kg of weight) currently participating the endurance competition were used. The field tests selected for the experiment was gallop (approximately 8.3 m/s) along the selected 2.5 km course (a natural forest trail, not artificial road; a closed loop course). The horses were divided into three groups according to their age; 3–4 years of age (G1, 3.29 ± 0.49 year), 6–7 years of age (G2, 6.42 ± 0.53), and 8–9 years of age (G3, 8.50 ± 0.55). The measurements times for the heart rate, blood lactate concentration, PCV, and Hb analysis were conducted before exercise (T0), shortly after exercise (T1), 15 min after exercise (T2), and 30 min after exercise (T3), respectively. Data was analyzed using an analysis of covariance (ANCOVA) for repeated measures with times and groups.

**Results:**

The results of the comparison depending on the passage of rest time after exercise suggest that the heart rate and blood lactate concentration of three groups at T2 significantly decreased compared to T1 (*p* < 0.001). PCV of the G2 and G3 groups were significantly decreased at T2 compared to T1 (*p* < 0.01). Hb values at G2 (*p* < 0.01) and G3 (*p* < 0.001) groups were significantly decreased at T2 as compared to T1. However, heart rate, blood lactate concentration, PCV and Hb level at T1 showed no difference in the comparison of horses from different age groups with the exception of G3 group in terms of heart rate.

**Conclusion:**

The physiologic and hematological responses of horses during recovery time after 2,500 m exercise with gallop were no significant difference among the groups. These data are useful as a response evaluation method for training of endurance horses.

## Background

Exercise-physiological research on horses is particularly meaningful in that it provides new information on how improve exercise performance [1]. The improvement of horses’ exercise performance is related to the improvement of cardiovascular system through exercise [2] and continuous training reduces exercise-related stressors by inducing adaptive responses [3].

As a result of exercise, treadmill and the field test method are usually used as standard exercise tests of the performance of horses [4]. As an exercise test with the maximum load of racehorses that is available by using a mechanical device, treadmill is widely used to evaluate the exercise capacity of racehorses [5–7]. Furthermore, the field test is standardized as a exercise test method and is used in evaluation of various horses; the strength of this method is that it can be conducted in a condition that is similar to the actual situation [6].

Numerous previous studies have addressed the issue of evaluating exercise capacity of horses [3, 8, 9]. Among other inspection items, the heart rate measurement for evaluating horses’ exercise capacity has been widely used as a useful method to determine the cardiovascular system capacity [10–12]. Also, since the blood lactate concentration is increased in proportion to exercise intensity before and after exercise, it is very useful for evaluation exercise intensity and training response [3, 13, 14]. In addition, it is possible to analyze exercise capacity and efficacy by analyzing the concentration of hemoglobin; this measurement makes it possible to identify the contraction of spleen and packed cell volume (PCV) that could predict the oxygen transportation capacity. These parameters provide meaningful information about fitness changes in horses [15].

In general, depending on various factors, such as experience, competition, training status, field or indoor, horses can show considerable individual differences [16]. Another important factor is horses’ age that may impact their cardiopulmonary function [17, 18]. For this reason, the performance assessment of horses in the context of various environmental factors is important in terms of evaluating health status and physical strength of horses [19, 20]. This is particularly true for horses used for endurance horse-riding, as these horses may be exposed to various parameters (weather, audience, decoration, topography, and etc.) during field horse-riding [16]. Therefore, it is very important to test the physiological change in these horses caused by field training or field competition [8].

Recently, as the horse industry development, the number of endurance riding competition has been increased. And also, riders are growing interest horse’s field training for participation competition. Physical exercise can change the physiologic metabolism of horses. This is because it can lead to changes in the blood constituents levels of horses. However, only a few studies have been reported on the evaluation of horse responses during a training. Therefore the purpose of this study was to investigate the physiologic and hematological response of horses during recovery time after a 2,500-m exercise with gallop.

## Methods

### Animals

All experimental procedures were approved by the IACUC (Institutional Animal Care and Use Committee) and Ethics Committee for Human Research (ECHR) of Jeju National University, South Korea. Twenty healthy 3–9 years-old Jeju crossbreed (Thoroughbred × Jeju native) mares (5.95 ± 2.24 year of age, 312.65 ± 13.59 kg of weight, and 140.50 ± 3.03 cm of height) currently participating the endurance competition were used.

The horse’s peak racing age has been reported between 4 and 5 years old [21, 22] and the age of horse can affect cardiopulmonary function [17]. On the basis of this, we divided into three groups in this study.

All horses that had participated in endurance competition (40 km) were used and were divided into three groups according to their age; 3–4 years of age (G1, 7 horses, 3.29 ± 0.49 year, 140.71 ± 3.64 cm, 317.14 ± 11.30 kg), 6–7 years of age (G2, 7 horses, 6.42 ± 0.53 year, 141.29 ± 2.81 cm, 315.00 ± 10.63 kg), and 8–9 years of age (G3, 6 horses, 8.50 ± 0.55 year, 139.33 ± 2.66 cm, 304.67 ± 17.33 kg). Horses were fed about forage 1.5% and mixture of forage 2.25% of their body weight in the same amount twice a day (06:00 and 16:00) and had free with automatic water system.

### Experimental design

Prior to the experiment, a pilot test was conducted to check if the course was gradual for gallop. The suitable location for the experiment was selected together with the riders that all were skilled experts. At that time, the horses that worked together with the riders had no relation with the experiment. The selected course was approximately 2.5 km long (a natural forest trail, not artificial road; a closed loop course), and the major exercise selected for the experiment was gallop (approximately 8.3 m/s) along the selected 2.5 km course. The starting and finishing points were in same place and ca. 200 m (40 × 50 m) circular track was constructed to conduct warming-up and closing exercise before and after the major exercise. The measurements times for the heart rate, blood lactate concentration, PCV, and Hb analysis were executed before exercise (T0), shortly (about within 1 min) after exercise (T1), 15 min (T2), and 30 min after exercise (T3). In previous research, a rapid change in physiological parameters of horses after high-intensity exercise showed a peak within 15 min, and the most effective way of recovery was trot [23]. Therefore, 10 min of trot in the circular track was carried out by every horse as a part of warming-up and closing exercise; the speed of trot was 3.8 m/s and 3.5 m/s for warming-up and closing exercise, respectively. The horses were running on the designated track after finishing the warming-up in the circular track prior to departing for the course; T1 was measured shortly after (about within 1 min) completion of exercise. After the measurement, trot was carried out as a closing exercise. The sequence of exercise was random departure one by one, and a new horse departed the starting line when the previous horse was at the distance of 500 ~ 600 m ahead of the finish line.

The riders were skilled experts (with over 5 years of riding experience and endurance competition experience). All riders were male and were aged between 37 and 42 years old (average age 38.17 ± 2.04 year, average weight 68.83 ± 2.93 kg, average height 175.5 ± 2.26 cm).

### Hematological and physiological parameters

Heart rate was measured with a T31 Polar transmitter (Polar Equine, Finland). Blood samples were typically taken from the mid area on the left side of the jugular vein and then immediately stored in a vacuum blood collection tube. Blood lactate concentration was measured by L-Pro lactate analyzer (LT-1710, ARKRAY Inc., JAPAN). PCV was analyzed using a microhematocrit reader (Hawksley Co. UK) and Hb was analyzed using Coulter counter (MEK-6450, Nihon Koden, Japan).

### Statistical analyses

Statistical analyses were carried out with the SAS software (Statistical Analysis Systems package 8.01, Cary NC, USA). Data was analyzed using an analysis of variance (ANOVA) for repeated measures using the general linear models procedure. Post hoc analysis was performed by Tukey’s test. All values were reported as means ± SD and the level of significance was <0.05.

## Results

### Physiological parameters

**Fig. 1:**
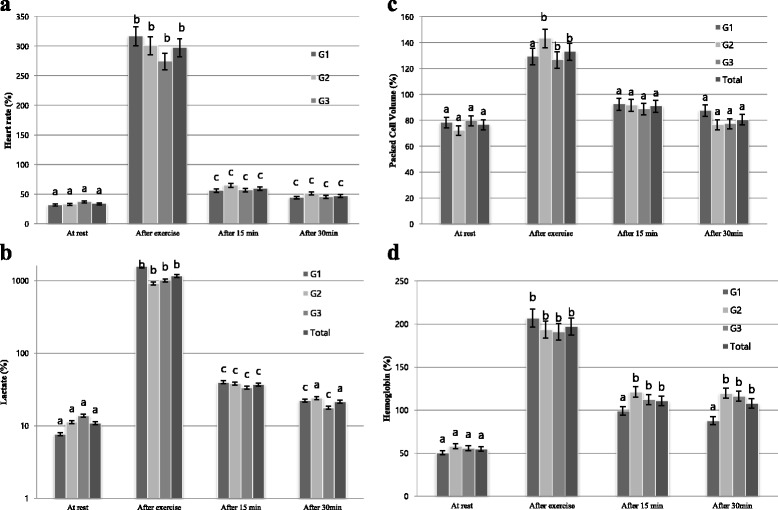
Mean values (%) of physiologic variables of horses during before and after exercise and recovery period. **a** Heart Rate; **b** Lactate; **c** Packed Cell Volume; **d** Hemoglobin

As concerns heart rate, significant differences were observed among the three tested groups at T0, T1 and T2; however, there was a significant difference at T2 and T3 (Table 1). In three groups, the heart rate significantly increased at T1 as compared to T0 (*p* < 0.001). The comparison of heart rate depending on the passage of rest time significantly decreased at T2 as compared to T1 in three groups (*p* < 0.001). The comparison of the increase of heart rate between groups at T1 showed the highest increase in G1 (*p* < 0.001) and the lowest increase in G3 (*p* < 0.001). From the comparison depending on rest time after exercise, the decrease of heart rate at T2 was in order of G1, G2, and G3, where G1 showed the fastest and G3 the lowest decreasing rate (Fig. 1a).

**Table 1:** Effect of age on physiologic parameters in field test of endurance horses

Items	Group	Experimental conditions (M ± SD)				Sig.
		T0	T1	T2	T3	
Heart Rate	G1	44.00^Aa^ ± 0.72	139.4^Ab^ ± 17.24	78.00^Ac^ ± 12.06	61.20^Ac^ ± 13.52	***
	G2	46.00^Aa^ ± 3.70	138.43^Ab^ ± 13.05	90.29^Ab^ ± 9.95	71.43^Ab^ ± 7.43	***
	G3	45.14^Aa^ ± 3.53	124.00^Ab^ ± 23.31	71.00^Bb^ ± 14.32	55.43^Ba^ ± 9.93	***
Lactate	G1	0.72^Aa^ ± 0.11	11.08^Ab^ ± 4.69	5.06^Aa^ ± 4.01	2.68^Aa^ ± 2.32	***
	G2	1.09^Aa^ ± 0.15	9.76^Ab^ ± 1.07	3.81^Ab^ ± 1.65	2.43^Aa^ ± 1.20	***
	G3	1.10^Aa^ ± 0.38	9.71^Ab^ ± 3.55	3.23^Aa^ ± 1.76	1.74^Aa^ ± 0.78	***

Means ± standard deviation, Levels of significance: ***P < 0.001

T0 = before exercise; T1 = shortly after exercise; T2 = after 15 min exercise; T3 = after 30 min exercise; G1 = 3–4 years of age; G2 = 6–7 years of age; G3 = 8–9 years of age

^A,B^Means with different superscripts in the same column significantly different (*P* < 0.05)

^a,b^Means with different superscripts in the same row significantly different (*P* < 0.05)

As concerns lactate concentration, no statistical differences were observed among the three groups from the comparison before and after exercise and rest time (Table 1). Lactate concentration significantly increased at T1 as compared to T0 in three groups (*p* < 0.001) and significantly decreased at T2 as compared to T1 (*p* < 0.001). The comparison of increasing rate of lactate concentration among the groups at T0 and T1 shows the highest increase in G1 and the lowest increase in G2. From the comparison depending on rest time after exercise, decreasing rate of lactate concentration at T2 was in order of G1, G3, and G2, where G1 showed the fastest and G2 the lowest decreasing rate (Fig. 1b).

### Hematological parameters

As concerns PCV, no statistical differences were observed among the three groups from the comparison before and after exercise and rest time (Table 2). PCV before and after exercise significantly increased at T1 as compared to T0 in G2 and G3 (*p* < 0.001) and increased in G1; however, this latter difference was not statistically significant. The comparison of PCV depending on the passage of rest time significantly decreased at T2 as compared to T1 (*p* < 0.001) in G2 and G3 and decreased in G1; however, this latter difference was not statistically significant. The comparison of the increase of of PCV among the groups before and after exercise showed the highest increase rate in G2 and the lowest increase rate in G3. From the comparison depending on rest time after exercise, the decrease of PCV at T2 was in order of G1, G2, and G3, where G2 showed the fastest and G1 the lowest decreasing rate (Fig. 1c).

**Table 2:** Effect of age on hematological parameters in field test of endurance horses

Items	Group	Experimental conditions (M ± SD)				Sig.
		T0	T1	T2	T3	
Packed Cell Volume	G1	39.40^Aa^ ± 4.51	50.40^Aa^ ± 3.65	46.20^Aa^ ± 5.89	43.60^Aa^ ± 8.91	NS
	G2	39.71^Aa^ ± 6.13	56.14^Ab^ ± 10.12	51.00^Aa^ ± 9.38	41.57^Aa^ ± 8.14	**
	G3	43.29^Aa^ ± 2.75	54.71^Ab^ ± 5.74	48.29^Aa^ ± 9.86	41.85^Aa^ ± 5.05	**
Hemo-globin	G1	12.06^Aa^ ± 2.43	24.60^Aa^ ± 6.29	23.58^Aa^ ± 7.72	20.82^Aa^ ± 10.38	NS
	G2	10.14^Aa^ ± 1.02	19.26^Ab^ ± 6.06	21.49^Ab^ ± 5.35	21.40^Ab^ ± 7.17	**
	G3	12.50^Aa^ ± 2.59	23.43^Ab^ ± 6.08	25.87^Ab^ ± 6.41	25.70^Ab^ ± 6.61	***

Means ± standard deviation, Levels of significance: NS, not significant; **P < 0.1; ***P < 0.001

T0 = before exercise; T1 = shortly after exercise; T2 = after 15 min exercise; T3 = after 30 min exercise; G1 = 3–4 years of age; G2 = 6–7 years of age; G3 = 8–9 years of age

^A,B^Means with different superscripts in the same column significantly different (*P* < 0.05)

^a,b^Means with different superscripts in the same row significantly different (*P* < 0.05)

As concerns, no statistical differences were observed among the three groups from the comparison before and after exercise and rest time (Table 1). Hb levels significantly increased at T1 as compared to T0 in G1 (*p* < 0.001), G2 (*p* < 0.001), and G3 (*p* < 0.001). The comparison of Hb depending on the passage of rest time significantly decreased at T3 as compared to T1 in all three groups, but there is no significant difference. The comparison of of the increase of Hb among the groups before and after exercise showed an increase in order of G1, G2, and G3 (Fig. 1d).

## Discussion

The results of the present study show that the physiological change level with regard to the recuperative power before and after a 2500-m high speed exercise and the passage of rest time did not differ among the three tested groups. Therefore, our results suggest that it is not necessary to make a big difference in exercise intensity and rest time in case of horses under the age of 3–9.

As concerns heart rate, heart rate is less than 42 beats per minute when the horse is in a stable status [6]. While heart rate may increase to 200 bpm in accordance with the intensity of exercise [24], once it exceeds the maximum heart rate, it does not increase though a fast exercise speed.

In general, 75 to 80% level of the maximum horses’ heart rate (HR max) is considered as the boundary of lactate threshold caused by anaerobic metabolism [13, 25]. In our study, the heart rate at T1 amounted to 124–139 bpm. This is not the heart rate level during exercise, but the one after exercise with which we can check whether anaerobic metabolism has occurred beyond lactate threshold during exercise [13, 25]. In order to provide appropriate care to a horse, it is essential to ensure its recuperative power after an anaerobic exercise. For this reason, not only the increase of heart rate depending on exercise intensity, but also the recuperation speed of a horse depending on passage of rest time is a useful parameter to evaluate the training status. If the ability of horse is improved, the heart rate decreases in the designated exercise speed or exercise intensity and decreases rapidly even after finishing exercise. In this study, horses’ average heart rate increased by 317% at T1 as compared to T0 and decreased by 272% at T3 as compared to T1 (*p* < .001).

Fatigue that is prone to occur by high-intensity exercise is caused by accumulation of lactate [26, 27]. Accumulation of lactate is used as an indicator of the causes of fatigue as a result of anaerobic exercise [28, 29] and the elimination of lactate means the relief of fatigue. In previous research, the effective cooldown exercise methods for a quick elimination of lactate concentration after high-intensity exercise in horses was reported [23]. Also a study on the cooldown methods for rapid removal of accumulated lactate concentration after swimming training, which is frequently used in racehorses, has been reported [30]. In these studies, the cooldown methods for the removal of lactate concentration accumulated after high intensity exercise reported rapid recovery after 15 min of rest in the order of trot, walk, and rest without exercise [23]. Accordingly, in the present study, in order to rapidly eliminate lactate, T2 was measured after executing 15 min of trot after the completion of major exercise as an aerobic exercise and T3 was measured again after 15 min of walk. As a result, blood lactate concentration at T1 significantly increased to 6.2-15.9 mmol/L as compared to T0 (*p* < .001); however G1 and G3 groups recuperated to the status before exercise at T2 and all three groups recuperated to the status before exercise at T3. This is thought to be a re-verification of Kang et al.’s [23, 30]. report that trot conducted immediately after exercise is effective for elimination of lactate. Lactate concentration in horses in a stable status is usually 1 mmol/L. Lactate concentration in horses in a stable status in the present study amounted to 0.72–1.10 mmol/L, which similar to the lactate concentration in a stable status. Therefore, the result of the present study can be judged to mean that there was no big excessiveness after exercise.

The higher the amount and intensity of exercise, the more the size of the heart, as the heart is adaptive. The higher the amount and intensity of exercise is increased the more the size of the heart, as the heart is adaptive. Oxygen absorbed from the lungs is transported to muscles through the blood and oxygen transportation capacity in horses means the speed of horse as much as that. Packed Cell Volume and Hb are measured to examine the change of oxygen transportation capacity in horses. Packed Cell Volume is recovered to T0 level from T2. However, Hb cannot be recovered to the status before exercise with only 30 min of rest. For example, Piccione et al. [14] investigated training and competition of standard-bred horses that did a 3-km exercise, which is similar to the distance used in the present study, with the exercise intensity conducted with a faster speed (750 m/min) than the one used in the present study (624 m/min). In our results, Hb level in horses in a stable status was 10.14 ± 1.02 ~ 12.50 ± 2.59; the same Hb level value (12.60 ± 0.94) was reported by Piccione et al. [14]. However, while, in Piccione et al. [14], Hb level shortly after exercise was 15.22 ± 1.78, it amounted to 22.20 ± 6.24 in our study, i.e. Hb level was higher level in spite of the slower exercise speed. However, in our results, Hb level at T3 showed 58% of decrease as compared to T1. Piccione et al. [14] reported Hb decreasing rate as 23% after 60 min of rest as compared to the one shortly after exercise. These differences between the results reported by [14] and the present study are considered to be largely affected by the environmental factors, such as shape of track, topography, type of actual exercise, breed of horses, and exercise career.

## Conclusions

The physiological change depending on the passage of rest time after high-speed exercise shows the most significant decrease at T2, which is 15 min after rest, as compared to T1. From the comparison between T1 and T2, heart rate, lactate concentration in blood, and Hb level significantly decreased at T2 as compared to T1, with the exception of PCV in G1 group. Furthermore, there was no difference among the tested groups in terms of heart rate, lactate concentration in blood, and Hb level, with the exception of PCV in G1 group from the comparison among the groups depending on the age of the horses. In case of T3, lactate concentration and PCV level recovered to the level before exercise, and heart rate and Hb level were found to have insufficient time to be recovered to the level before exercise. The recovery rate of horses depending on the passage of rest time after high-speed exercise is a crucial indicator, as this directly relates to the training status of horses. Therefore, we assume that difference in exercise intensity is not necessary for horses under the age of 3 ~ 9 years old. However, we considered that the efforts to reduce the fatigue level through the adaptation training to anaerobic exercise and to recover the homeostasis as quickly as possible after exercise will play a pivotal role in the preparation of next endurance.

## Abbreviations

G1: 3–4 years of age
G2: 6–7 years of age
G3: 8–9 years of age
Hb: Hemoglobin
PCV: Packed cell volume
T0: Before exercise
T1: Shortly after exercise
T2: After exercise 15 min
T3: After exercise 30 min
